# Thermochemical Degradation of a Polyacrylamide Gel as a Dual-Function Strategy for Enhanced Oil Recovery and Reservoir Remediation

**DOI:** 10.3390/gels11110915

**Published:** 2025-11-16

**Authors:** Jiaying Wang, Renbao Zhao, Yuan Yuan, Yunpeng Zhang, Guangsen Zhu, Jingtong Tian, Haiyang Zhang, Haitao Ren, Guanghui Zhou, Bin Liao

**Affiliations:** State Key Laboratory of Petroleum Resources and Engineering, China University of Petroleum (Beijing), Beijing 102249, China; wangjiayingswpu@163.com (J.W.); yuany9939@163.com (Y.Y.); 2024215221@student.cup.edu.cn (Y.Z.); 2023210410@student.cup.edu.cn (G.Z.); 2023210409@student.cup.edu.cn (J.T.); 2022215216@student.cup.edu.cn (H.Z.); 15935903697@163.com (H.R.); zhouguanghui0603@163.com (G.Z.); 18079745126@163.com (B.L.)

**Keywords:** polymer gel-treated reservoirs, in-situ degradation, thermochemical conversion, activation energy reduction, reservoir remediation, oil recovery

## Abstract

The accumulation of residual hydrolyzed polyacrylamide (HPAM) gel or molecular-based solutions in reservoirs after polymer flooding poses dual challenges: irreversible formation damage and long-term environmental risk issues. However, existing research mainly focuses on treating polymers in surface-produced water, neglecting both in situ decomposition of residual polymer gel or molecular-based solutions in reservoirs and the degradation of HPAM gels under high temperatures from in situ combustion (ISC). This work investigates the thermochemical behavior of HPAM gel during ISC and its dual-function role in enhanced oil recovery (EOR) and reservoir remediation. It was demonstrated that the residual gel and/or molecular-based solutions undergo efficient degradation, serving as an in situ fuel that significantly reduces the activation energy for crude oil oxidation by up to 58.4% in the low-temperature stage and 75.2% in the high-temperature stage. Factors influencing the gel’s degradation and the combustion process, including its molecular weight, ionic type, and crude oil viscosity, were systematically evaluated. Optimal conditions achieved over 90% gel degradation. Combustion tube experiments validated the dual benefits of this approach: an incremental oil recovery of 68.6% and an average HPAM gel removal efficiency of 64.8%. This work presents a novel strategy for utilizing retained gels in situ to simultaneously enhance oil recovery and mitigate gel-induced formation damage, offering significant insights for the management of mature gel-treated reservoirs.

## 1. Introduction

Polymer flooding [1,2,3] is considered a mature enhanced oil recovery technique designed to improve sweep efficiency over waterflooding, where the adverse viscosity ratio ($\mu_{o}/\mu_{w}$) between oil and water leads to unstable displacement [4]. The polymer flooding method can further enhance the oil recovery factor and has shown remarkable performance in many industrial-scale applications as the oil field is developed into a high water cut period, which is mainly attributed to its better mobility control during the displacement process [5]. The viscous polymer solution could significantly reduce the viscous fingering phenomenon, allowing the displacing fluid to advance more evenly in the reservoir pores. Therefore, more sweep efficiency is achieved, accompanied by more crude oil recovered. The technical and economic success of polymer flooding has led to its widespread global implementation, with over 70 field projects currently operational worldwide, particularly in China, Canada, and Oman, demonstrating incremental recoveries of 5–20% STOIIP [6,7,8,9,10]. HPAM has been the most commonly applied polymer in the field [11]. Recent research has shown that modifying polyacrylamide with sodium p-styrenesulfonate (SSS) can effectively enhance its high-temperature resistance—exhibiting excellent stability even at 180 °C—by increasing the gyration radius, reducing the diffusion coefficient, stabilizing hydrogen bonds, and optimizing the spatial network structure (e.g., widened branches and regulated pore parameters) [12]. This modification provides a new approach for improving polyacrylamide’s adaptability to deep high-temperature reservoirs, addressing the degradation issue of conventional polyacrylamide under extreme thermal conditions.

**Figure 1:**
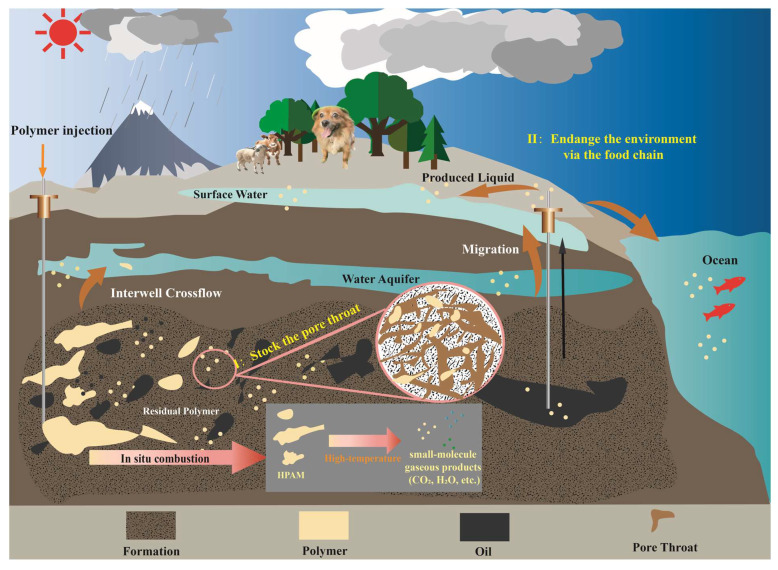
Schematic diagram of potential ecological risks of underground residual HPAM and proposed solution pathways.

However, with the continuous injection of polymer solution or gels, especially in the later stage of polymer EOR, serious problems have gradually emerged [13]. Long-term injection of polymer gels decreases the effective permeability of the reservoir and plugs the formation pores, resulting in irreversible reservoir damage [14,15]. Specifically, in the industrial polymer injection blocks of the Daqing Oilfield, the polymer gel retention rate was found to be in the range of 70% to 80% at the end of polymer injection. Subsequently, after 5 to 6 years of water flooding, the polymer gel residue rate was observed to have decreased by 12.8% to 17.6%. However, it should be noted that 60% of the polymer is still retained underground [16]. Moreover, in Block II4-5 of the Shuanghe North Fault Block in the Henan Oilfield, a total of 3628.48 tons of polymer has been injected into the oil reservoirs through prepared solutions of different concentrations with surface and/or produced formation water. When the subsequent water flooding was carried out for 10 months, the amount of polymer remaining in the formation was measured to be 3103.80 tons, and correspondingly, with a retention rate of 85.54% [17]. Critically, retained polymers pose substantial environmental risks. Following the injection of polymers into reservoirs for enhanced oil recovery, a portion of the polymers becomes retained through mechanisms, including adsorption, thereby blocking pore throats. Another fraction may migrate to adjacent aquifers due to interlayer crossflow, while the remainder is produced alongside oil–water effluents at production wells (Figure 1). After polyacrylamide (PAM) enters the subsurface environment, its ecological risks mainly stem from the migration and diffusion of residual acrylamide monomer (AM) in the product. Acrylamide is a carcinogenic, mutagenic, and reprotoxic monomer [18]. It can cause biological damage by affecting the central nervous system. Subsurface AM can enter groundwater along with partially hydrolyzed polyacrylamide from wellbore leakage or interlayer migration. AM in groundwater has extremely low reactivity, which is verified when no significant biodegradation occurs, and sandy sediments have difficulty adsorbing it, allowing it to exist stably for a long time [19]. Therefore, it would endanger aquatic organisms dependent on groundwater, which may have a serious impact on their growth, reproduction, and survival [20,21], thereby affecting the surface ecosystem through the food chain.

The natural degradation of synthetic polymers exhibits exceptionally prolonged durations spanning centuries, primarily due to the absence of microbial degradation mechanisms, inherent molecular structural resistance [22], and constraints imposed by oxygen-deprived environmental conditions. Particularly within oil reservoir formations, these polymers persist without complete conversion into environmentally neutral substances. Consequently, during this extended period, the detrimental impact of residual HPAM gel on the environment persists. Polymer flooding achieves only limited enhancement in oil recovery due to inherent constraints in sweep efficiency modification. This restricted effectiveness leaves substantial residual hydrocarbon resources retained within the polymer-flooded oil reservoirs, maintaining significant potential for subsequent extraction through other recovery strategies. However, the residual polymers seriously decrease the injection capacity and trigger great challenges for further exploitation during the subsequent reservoir development period of crude oil, and they pose a detriment to the substance environment. Nevertheless, currently, the negative impacts of residual polymers have not been paid sufficient attention. Therefore, the removal of underground polymers remains a topic that has been scarcely explored, with few related studies so far.

ISC [23,24,25] is one of the thermal EOR methods that could be applicable in a variety of reservoirs. Air or other oxygen-enriched gas is injected into the formation and ignited during the process. Heavy components undergo complex thermal cracking and oxidation reactions, including low-temperature oxidation (LTO), fuel deposition (FD), and high-temperature oxidation (HTO), generating a huge amount of heat as well. Propelled by the heat and produced gases, the crude oil is efficiently displaced and recovered, thereby enhancing the oil recovery. In this work, ISC is proposed as a method that can both enhance oil recovery and remove the underground residual polymers. The high-temperature environment causes the polymers to be degraded or even burned, thereby restoring part of the reservoir permeability and improving the seepage performance of the reservoir. At the same time, a large amount of gas generation could increase the internal pressure of the reservoir and drive the crude oil to flow towards the production well, thereby achieving a higher recovery factor. Therefore, it is of great significance to conduct experimental research on the oxidation characteristics of polymers during the ISC process. It helps to deeply explore the potential and feasibility of ISC in removing the remaining underground polymers.

Currently, there has been a certain amount of research on the pyrolysis behaviors of polyacrylamide [26,27]. However, most of these studies have been focused on the pyrolysis process in bulk or simple systems, lacking in-depth analysis of the oxidative pyrolysis of HPAM gel in porous media. Self-designed reaction equipment was utilized in this work. A comprehensive investigation was carried out into the oxidation and pyrolysis characteristics exhibited by HPAM gel within porous media. The activation energy of various samples during the combustion process was quantitatively calculated. Furthermore, the feasibility of the degradation and removal of underground residual polymer gel during the ISC process was explored. In addition, an in-depth examination was also conducted to clarify the impact of the molecular weight, the ionic type of the gel, and the viscosity of crude oil throughout the ISC process. Moreover, the conductivity method was proposed to determine the content of HPAM gel in oil sand, so as to ascertain the degradation efficiency of HPAM gel under various conditions.

While extensive research has been reported on polymer gel and/or molecular-based solution applications in EOR, critical gaps remain in using in situ degradation to achieve both reservoir remediation and incremental recovery. Previous studies mostly focus on chemical degradation mechanisms under ideal conditions or isolated evaluations of EOR performance. None, however, explores the synergetic potential of turning trapped polymer into agents that boost recovery. This work demonstrates that ISC can effectively remediate residual polymer gel and/or solution contamination in post-polymer-flooded reservoirs. The experimental results validate the ISC method as a promising solution for addressing both polymer gel-induced formation damage and groundwater contamination in mature polymer-treated reservoirs. Suggesting the ISC technique could not only enhance oil recovery but also provide an environmentally sustainable approach to reservoir remediation.

## 2. Results and Discussion

### 2.1. The Oxidation Behavior and Residues of HPAM

**Figure 2:**
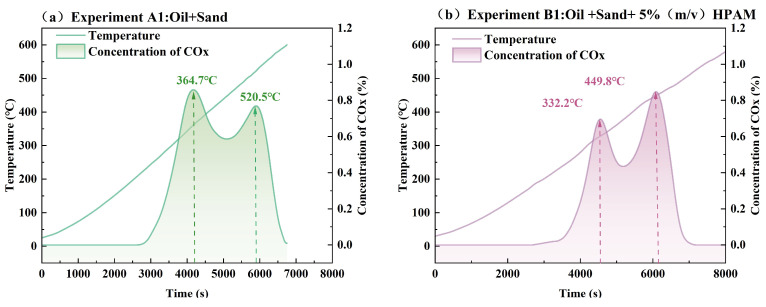
Temperature and concentration of COx variations in Experiment A1 (**a**) and B1 (**b**).

During the kinetic cell experiment, real-time monitoring and recording were meticulously implemented to track the variations in temperature and the changes in the concentration of the effluent gases. The variation in carbon dioxide (COx) concentrations and temperature is widely used as a key indicator for monitoring the reaction progress of ISC and polymer pyrolysis [26,28]. They were finally analyzed and graphically represented in Figure 2, highlighting distinct combustion stages and their associated reaction characteristics.

Figure 2 illustrates the temperature and concentration of COx variations in Experiments A1 (a) and B1 (b) under identical conditions. Both profiles reveal dual COx concentration peaks associated with LTO and high-temperature HTO processes. The first peak (364.7 °C in A1 vs. 332.2 °C in B1) corresponds to low-temperature oxidation, where heavy hydrocarbons undergo sequential reactions including vaporization, oxygen addition, and peroxide formation, generating coke deposits alongside CO_X_ emissions. The subsequent peak (520.5 °C in A1 vs. 449.8 °C in B1) arises from high-temperature oxidation, characterized by thermal cracking of residual hydrocarbons into CO_X_ and H_2_O through exothermic pathways.

The combustion process in both experiments exhibited exothermic behavior, characterized by distinct temperature and COx concentration profiles. Notably, Experiment B1 demonstrated significantly enhanced exothermicity, as evidenced by a hump in temperature elevations at both COx peaks compared to Experiment A1. This suggests that HPAM gel amplifies the heat release during the combustion process. Furthermore, the presence of HPAM resulted in a notable reduction in peak temperatures: the first COx peak occurred at 332.2 °C in B1, 32.5 °C lower than the 364.7 °C observed in A1, while the second peak shifted from 520.5 °C in A1 to 449.8 °C in B1, a reduction of 70.7 °C. It indicates that the HPAM gel alters the combustion kinetics, likely by lowering the activation energy required for oxidation reactions, which will be described in Section 2.2.1.

**Figure 3:**
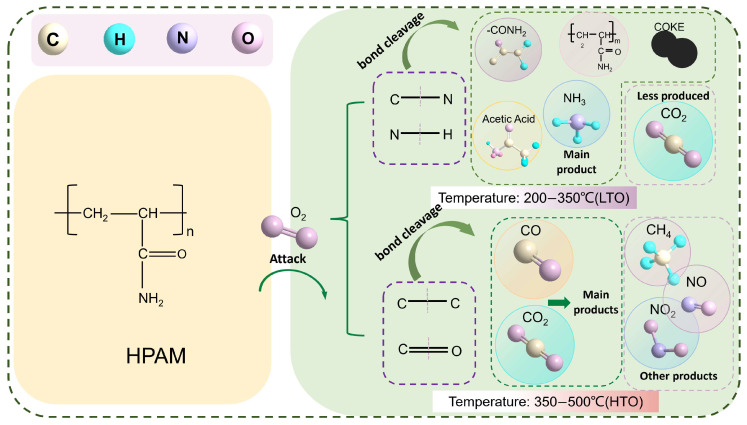
HPAM molecular evolution in different temperature intervals.

These findings suggest that the residual HPAM gel within the porous media reduced both the low-temperature oxidation and high-temperature oxidation thresholds, with a more pronounced decrease in the HTO stage. This is likely due to nitrogen-containing radicals (e.g., –NH_2_) from HPAM pyrolysis lowering the oxidation activation energy and accelerating reactions, while enhanced radical activity at high temperatures significantly shortens the induction period. Figure 3 illustrates a possible evolution mechanism of HPAM gel during this process. In the LTO range, the C–N and C–C bonds in the side chain break first due to their relatively low bond energies. The primary products at this stage are ammonia, amide groups, and a small amount of carbon dioxide. As the temperature rises to the HTO range, the C–C bonds in the backbone and C=O bonds, as well as N–H bonds, undergo cleavage, generating large quantities of carbon oxides and short-chain alkanes such as methane. Additionally, the exothermic decomposition of HPAM itself releases heat, further promoting oxidation and synergistically reducing both LTO and HTO temperatures, which is also consistent with a previous experimental study on the pyrolysis of polyacrylamide [29,30].

**Figure 4:**
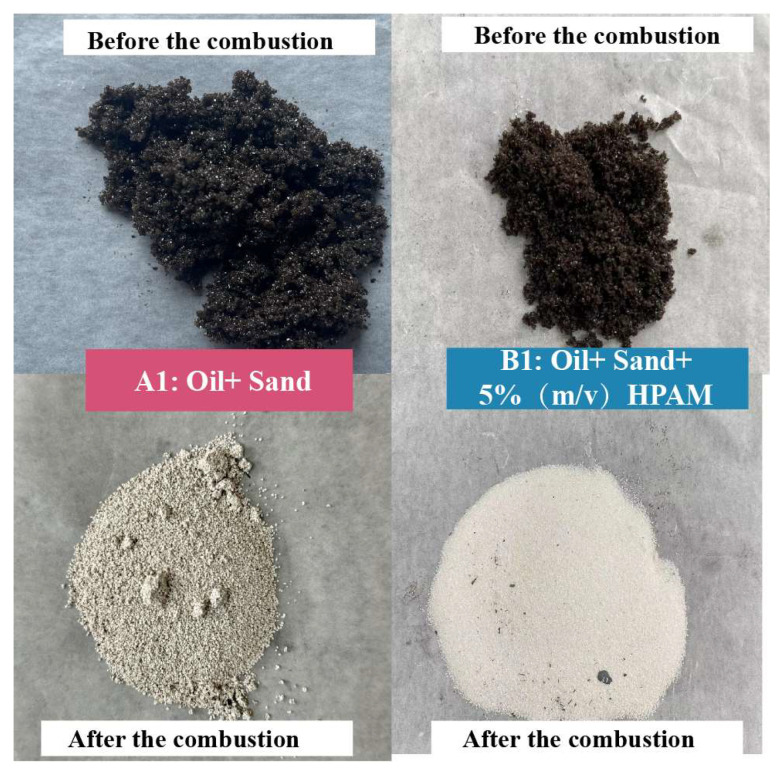
Appearance of samples in A1 and B1 before and after combustion.

Figure 4 shows the visual characteristics of the samples in Experiments A1 and B1 before and after the combustion. The oil sand in A1 exhibited a dark brown color before combustion. Samples were finally retrieved from the reactor upon ending the experiment.

It was distinctly discernible that the sample from Experiment A1 had turned white after combustion, signifying the complete combustion of the crude oil with no residual oil. In contrast, the sample from Experiment B1 manifested an off-white appearance after combustion. This phenomenon could potentially be ascribed to the fact that during the experimental process, a small amount of coke, which was generated as a byproduct of HPAM decomposition, remained entrapped within the oil sand matrix.

The degradation efficiency of HPAM gel in Experiment B1 was calculated to be 86.7%. This result indicates that the residual HPAM gel in the oil sand can be effectively degraded during the ISC process. This finding confirms that ISC can substantially remove residual polymers in reservoirs that have undergone polymer flooding, thereby mitigating issues related to polymer clogging and contamination, offering a dual benefit of enhanced oil recovery and environmental cleanup.

### 2.2. Oxidation and Degradation Characteristics of HPAM Gel Under Different Porous Media

Previous studies have confirmed that residual polymer gels retained in porous media are not only combustible but also exhibit a degree of enhancement for in situ combustion processes in oil reservoirs. Subsequently, a detailed investigation of the impact of residual polymers in porous media on the activation energy of crude oil during the oxidation reaction process was carried out. The potential role of HPAM gel as a sacrificial reaction promoter in the in situ combustion process was further quantitatively evaluated. Meanwhile, oxidation behaviors of HPAM gel with distinct molecular properties and crude oils of varying viscosities were compared under controlled in situ combustion conditions.

#### 2.2.1. Influence of the HPAM Gel on the Active Energy of the Oil

**Figure 5:**
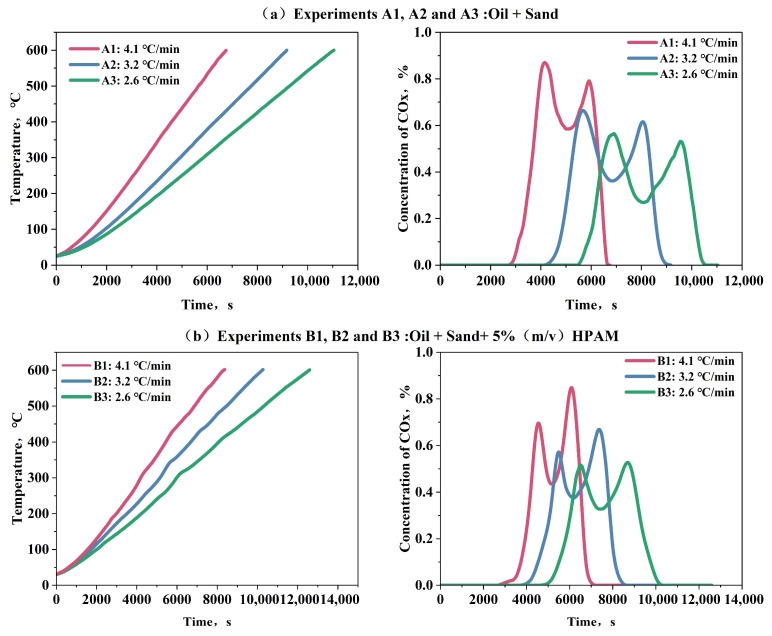
Temperature and concentration variations in Experiments A1 to A3 (**a**) and B1 to B3 (**b**).

Figure 5 illustrates the temperature profiles and COx concentration trends observed during Experiments A1–A3 and B1–B3, providing insights into the oxidation dynamics and the influence of HPAM gel on reaction kinetics.

**Figure 6:**
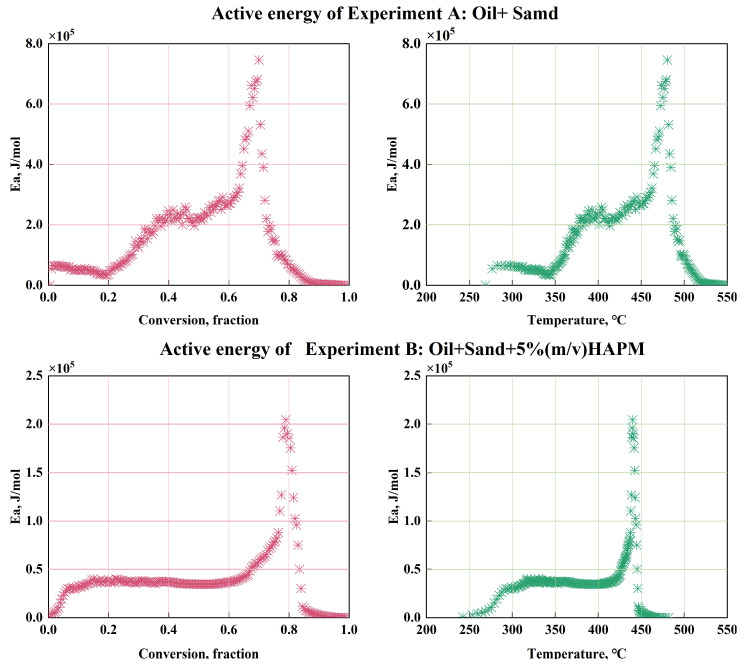
Activation energy fingerprints in groups A and B.

A comparison in Figure 5 reveals that in the latter, each set of temperature variation curves exhibits two distinct peaks corresponding to the two peaks in concentration variation. This indicates a more pronounced exothermic reaction in the oil sand containing HPAM gel within the porous medium. Additionally, the carbon oxide concentration variations across all six experiments exhibit two peaks, which can be associated with the LTO and HTO processes of the oil. Specifically, the first peak in Group A is higher than the second, whereas the second peak in Group B is elevated. This suggests that HPAM gel primarily promotes the HTO process, leading to increased carbon oxide production during HTO compared to LTO. Using the Friedman method, the activation energies of Groups A and B were calculated and presented in Table 1 and Table 2, respectively, with their activation energy fingerprints shown in Figure 6.

Analysis of the activation energy data for Group A reveals that a conversion rate ranging from 0.005 to 0.27 corresponds to a temperature interval of 268.7–359.5 °C, aligning with the LTO stage where oxygen addition and cracking reactions are dominant. Here, activation energy ranges from 33.213 to 96.475 kJ/mol. At conversion rates between 0.27 and 0.58 (359.5–445.1 °C), crude oil cracking reactions become dominant, with activation energies ranging from 99.612 to 291.423 kJ/mol. When the conversion rate exceeds 0.89, the crude oil enters the high-temperature oxidation stage (445.1–523.5 °C), with activation energies ranging approximately from 291.423 to 745.637 kJ/mol.

Compared with Group A, the activation energy results in Group B reveal significant reductions in both LTO and HTO processes. The maximum activation energy of low-temperature oxidation is diminished from 96.475 kJ/mol to 40.125 kJ/mol, with a reduction rate of 58.4%. The maximum activation energy in the high-temperature oxidation stage is lowered from 745.637 kJ/mol to 185.124 kJ/mol, with a reduction rate of 75.2%. This promoting effect can be attributed to the heat released during HPAM gel decomposition, which facilitates the oxidation process and reduces the activation energy required for crude oil oxidation. These findings provide quantitative evidence of the gel’s promoting role in the ISC process.

#### 2.2.2. Oxidation Characteristics of HPAM Gel with Different Properties

To investigate the influence of HPAM gel properties on the ISC process of crude oil, six sets of experiments were conducted. Experiments C1, C2, and C3 were designed to study the effect of molecular weight on oxidation behavior in simulated oil sand reservoirs, with each experiment utilizing HPAM of different molecular weights. Experiments D1, D2, and D3 focused on the impact of HPAM ionic types, specifically cationic polyacrylamide, anionic polyacrylamide, and nonionic polyacrylamide, respectively.

1. Oxidation characteristics of HPAM gel with different molecular weights

**Figure 7:**
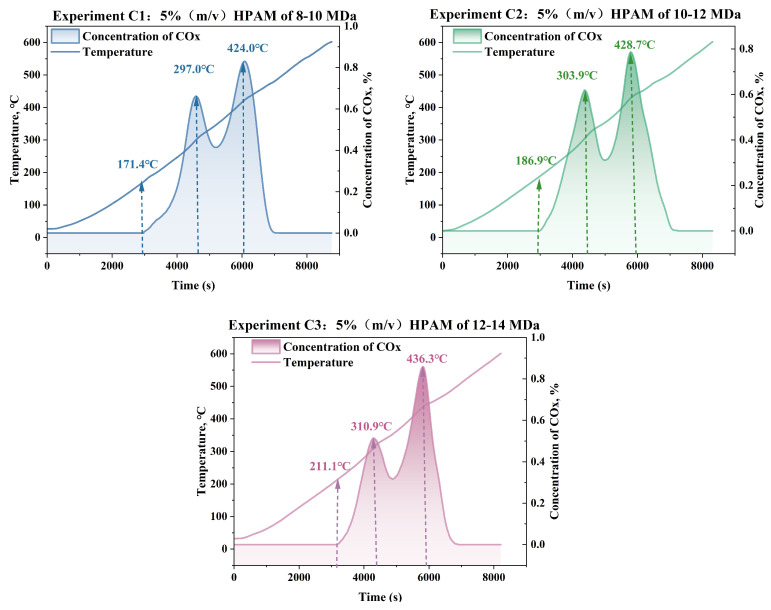
Temperature and concentration profiles of Experiments C1–C3 with HPAM of varying molecular weights.

Figure 7 presents the experiment results of the mixed samples of oil sand with HPAM of three different molecular weights (Experiments C1, C2, and C3). Each set of experimental results is presented in the form of curves showing the variations in temperature and COx concentration over time.

It is observed that as the molecular weight of HPAM increases, the temperature required to generate COx also rises. Specifically, in Experiment C1 with the HPAM of molecular weight ranging from 8 to 10 MDa, COx production began at approximately 171.4 °C. For Experiment C2, with an HPAM of 10–12 MDa molecular weight, this initiation temperature increased to 186.9 °C. In Experiment C3, with HPAM with a molecular weight of 12–14 MDa, the temperature further rose to 211.1 °C. A similar trend was observed for the first and second COx concentration peaks. The first peak temperature increased from 297.0 °C in C1 to 303.9 °C in C2 and 310.9 °C in C3. The second peak temperature showed an even more pronounced increase, rising from 424.0 °C in C1 to 436.3 °C in C3. These trends can be attributed to the structural properties of higher-molecular-weight HPAM, which feature longer molecular chains, tighter entanglement, and stronger intermolecular forces. As a result, more energy is required to break and degrade these chains, leading to higher initiation and peak temperatures.

**Figure 8:**
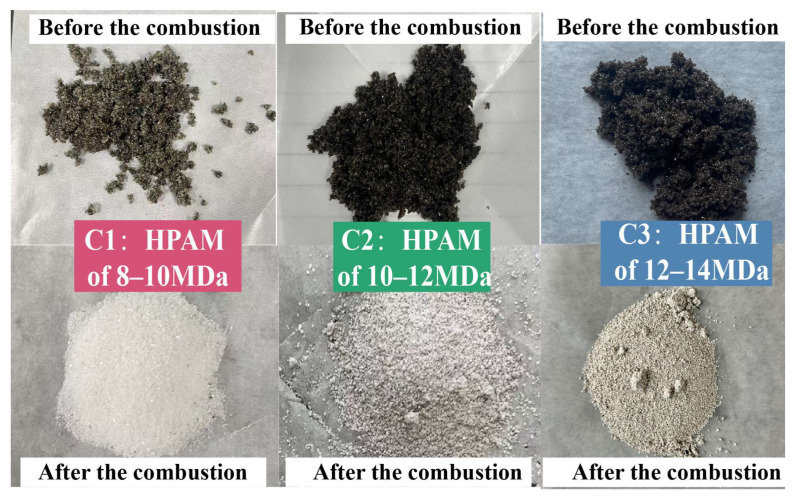
Appearance of samples from Experiments C1, C2, and C3 before and after combustion.

These results indicate that the lower-molecular-weight HPAM gel has a more pronounced promoting effect on the ISC process of crude oil. Its simpler molecular structure facilitates easier chain breakage and degradation, making it more effective at promoting oxidation and reducing reaction temperatures. Figure 8 exhibits the appearance of the samples from Experiments C1, C2, and C3 before and after combustion. All three samples exhibited similar appearances, displaying white or off-white coloration, which indicates complete or near-complete degradation of the hydrocarbons and HPAM gel. The degradation efficiencies of the samples were measured based on the conductivity method described earlier, and the test results are shown in Table 3.

The results demonstrate that samples with different molecular weights of HPAM gel undergo significant degradation during the ISC process in porous media. There is a clear inverse relationship between the degradation efficiency and molecular weight. That means that when the molecular weight increases, the degradation rate decreases. For example, when the molecular weight of HPAM increases from between 8 and 10 million to between 12 and 14 million, the degradation rate has a noticeable drop, falling from 91.5% to 86.7%. This shows that the higher the molecular weight, the more difficult it is for the HPAM gel to be degraded under the same treatment conditions. The possible reason is that high-molecular-weight HPAM has longer molecular chains, more complex structures, and more stable chemical bonds, requiring higher energy or more severe conditions to trigger its degradation reaction, thus leading to the decline in the degradation rate. Consequently, residual gel with a lower molecular weight of HPAM in the reservoir is more susceptible to cracking during the ISC process, making it a more effective candidate for enhancing oxidation efficiency.

The result is consistent with the research of Shatat [31]. In their work, it was confirmed that high-molecular-weight HPAM has a more stable structure and relatively lower degradation efficiency compared with low-molecular-weight HPAM.

2. Oxidation characteristics of HPAM gel with different ionic types

**Figure 9:**
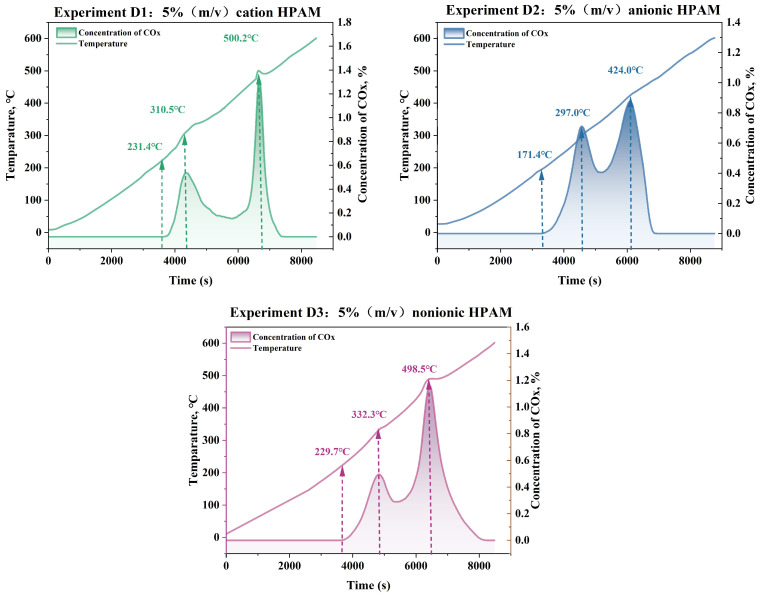
Temperature and concentration profiles of Experiments D1–D3 with varying types of HPAM.

Gel with different ionic types of HPAM is expected to have different degrees of influence on the ISC process of crude oil. Figure 9 presents the temperature and concentration curves of HPAM with cationic (Experiment D1), anionic (Experiment D2), and nonionic (Experiment D3) properties in porous media, illustrating how these structural variations affect the oxidation behavior.

It shows that anionic HPAM gel exhibits the lowest degradation initiation temperature (171.4 °C) during the ISC process, whereas cationic and nonionic HPAM gel require higher initiation temperatures (231.4 °C and 229.7 °C, respectively), indicating greater energy requirements for reaction activation. While anionic HPAM (D2) exhibits the earliest degradation in porous media, its COx concentrations show comparable levels between LTO (0.7%) and HTO (0.5–0.9%). In contrast, cationic (D1) and nonionic HPAM (D3) display pronounced promotion selectivity: their HTO peaks (1.2–1.4%) surpass LTO peaks (0.5%) by 140–180%, indicating superior high-temperature promotion efficiency despite requiring higher initiation temperatures.

This can be attributed to the cationic groups with positive charges on the molecular chain of cationic HPAM, which strengthen intermolecular forces, making molecular chain movement relatively difficult and thus enhancing thermal stability to some extent, leading to a relatively high degradation temperature. Conversely, the polar groups in the anionic HPAM molecular chain increase polarity and water absorption, predisposing it to hydrolysis and molecular chain breakage at high temperatures. For nonionic HPAM gel, the lack of ionic groups results in relatively weak intermolecular forces and a relatively active amide group. Consequently, the thermal stability of anionic and nonionic HPAM gel is relatively low, and so are their degradation temperatures.

**Figure 10:**
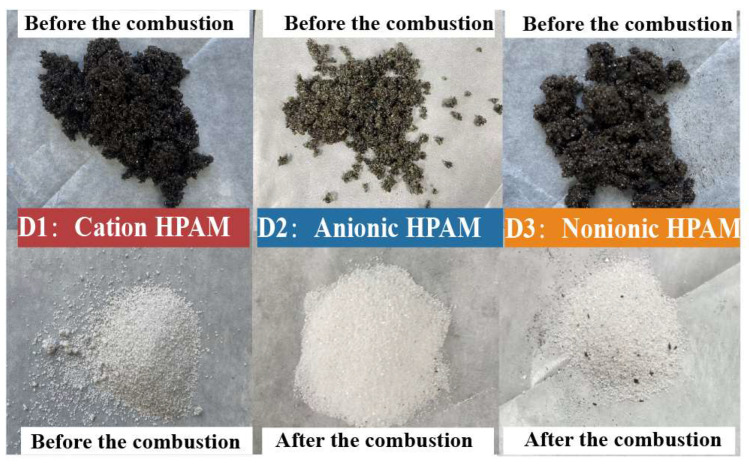
Appearances of samples in D1, D2, and D3 before and after combustion.

Similarly, a comparison of the appearances and degradation efficiency measurements was carried out on the samples of Experiments D1, D2, and D3 after oxidation, as shown in Figure 10 and Table 4.

Post-oxidation analysis revealed comparable visual characteristics across all three sample groups (D1, D2, and D3), with degradation efficiencies of 88.4%, 89.1%, and 87.9%, respectively. These results demonstrate that the residual HPAM gel could achieve degradation efficiencies higher than 85% in oil reservoirs during the ISC process, with the ionic type (anionic/cationic/nonionic) showing <1.5% variation in degradation outcomes under identical thermal conditions. The consistent degradation performance across HPAM variants suggests that ISC effectively mitigates polymer retention issues regardless of molecular charge characteristics, providing a robust remediation strategy for polymer gel-treated reservoirs.

Results show that differences in molecular weight resulted in a variation in degradation efficiency of up to 5%, whereas differences in ionic type led to a remarkably small variation of less than 1.5%. This comparison underscores that molecular weight serves as the primary controlling factor for HPAM degradation, to a greater extent than ionic type. The difference can be attributed to the distinct underlying factors each property influences. Molecular weight primarily governs the overall structural entanglement and thermal stability of the polymer chains, thereby dominating the fundamental difficulty of degradation and the ultimate efficiency. In contrast, the ionic type introduces different functional groups into the side chains. The varying thermal stability and reactivity of these specific functional groups are responsible for the differences observed in the initial pyrolysis temperatures. These findings are consistent with those reported in the literature [26].

However, once the temperature exceeds a critical threshold (e.g., upon entering the high-temperature oxidation stage above approximately 500 °C), all HPAM types undergo backbone scission and deep oxidation. The ISC process provides a high-temperature, oxygen-rich environment with abundant radical activity. Under these severe conditions, initial decomposition differences are overridden and all polymer structures are extensively oxidized, leading to consistently high degradation efficiencies exceeding 85% across all HPAM types investigated.

#### 2.2.3. Influence of Oil Viscosity on the Degradation and Oxidation of HPAM Gel

**Figure 11:**
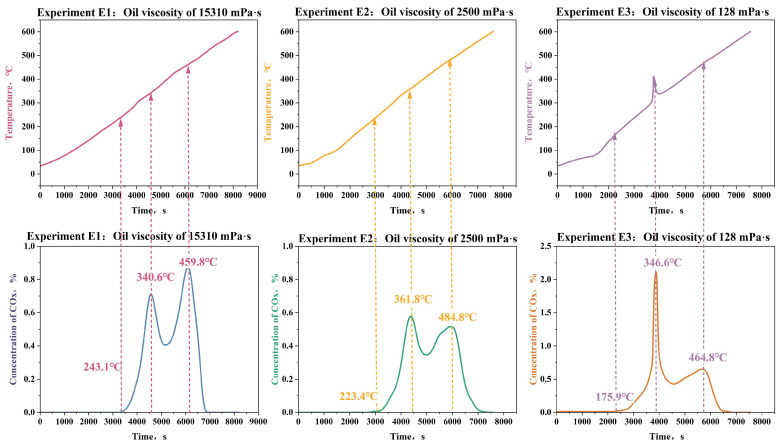
Temperature and concentration profiles of Experiments E1–E3 with oil of varying viscosities.

Figure 11 shows the temporal evolution of temperature and COx concentration profiles of E1, E2, and E3 containing crude oils of varying viscosity (128–15,310 mPa·s).

Analysis of reaction initiation temperatures reveals a positive correlation between crude oil viscosity and LTO thresholds: lower-viscosity oils (128 mPa·s) presented more activity with a lower initiation oxidation temperature of 175.9 °C, while higher viscosity oils require an oxidation temperature of 243.1 °C, which is much higher.

Additionally, when compared with the higher-viscosity crude oil, another notable difference emerges in the porous media environment of the lower-viscosity crude oil. Lower-viscosity mixtures exhibit distinct reaction behavior, showing that the COx concentrations during the low-temperature oxidation process are 2.1–3.5 times higher than those during the high-temperature oxidation process. This difference can be attributed to the fundamental properties of different oils. Light oil normally possesses a lower flash point (around 175 °C) and also a much lower ignition point (around 300–320 °C) than heavy oil in bulk conditions, which are presented in the ranges of 220–240 °C and 320–340 °C, respectively. The coke combustion temperature or HTO process, however, shows less of a difference, varying in the range of 460–480 °C for different oils.

Subsequently, post-oxidation analysis of Experiments E1–E3 shows HPAM gel degradation efficiencies quantified through conductivity measurements, which are shown in Table 5.

As the results indicate, there is a positive correlation between the crude oil viscosity and the degradation efficiency of HPAM gel in the system. Oil sand mixtures with higher viscosity demonstrate superior degradation performance, with E1 (15,310 mPa·s) achieving 93.1%, compared to 85.2% in E3 (128 mPa·s). The result could be attributed to the higher content of heavy components (e.g., asphaltenes and resins) in high-viscosity crude oil. During the oxidation, these heavy components preferentially form coke deposits, which extend the duration of high-temperature oxidation, thereby providing sustained thermal energy for HPAM molecular chain scission. Simultaneously, active free radicals (e.g., ·OH and O^−^) generated from heavy component oxidation could initiate oxidative degradation of HPAM backbones through thermo-chemical synergy. Furthermore, the higher calorific value of viscous crude oil may enhance energy supply efficiency during the oxidation reaction, further facilitating polymer decomposition. Notably, these findings highlight the potential advantage of high-viscosity crude oil in effectively removing HPAM contaminants during the ISC processes, offering critical insights for optimizing polymer pollution remediation strategies in heavy oil reservoirs.

This result indicates that during the in situ combustion process of crude oil with different viscosities, HPAM gel can be degraded to a large extent, ranging from 85.2% to 93.1%. The higher the viscosity of the crude oil, the more conducive it is to the degradation of HPAM during the ISC process.

### 2.3. Analysis of Enhanced Oil Recovery

**Figure 12:**
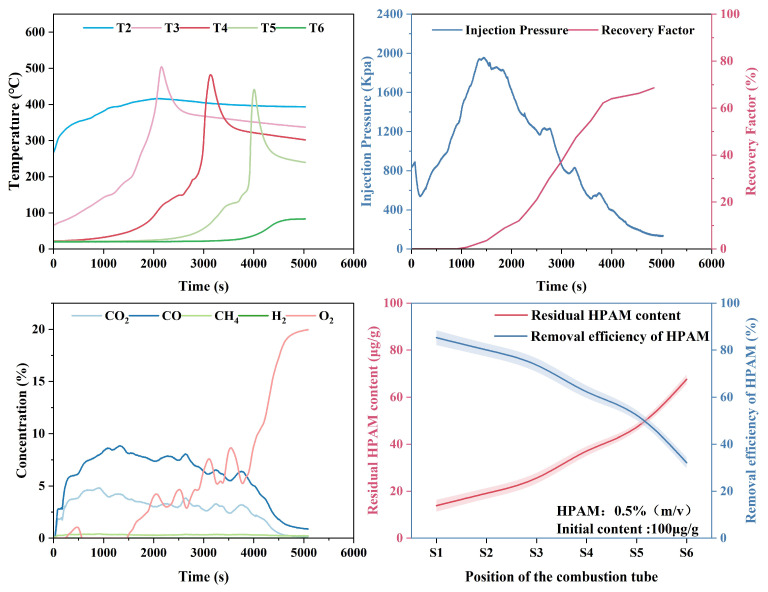
Results of combustion tube experiments: temperature, pressure, recovery factor, and HPAM gel degradation.

**Figure 13:**
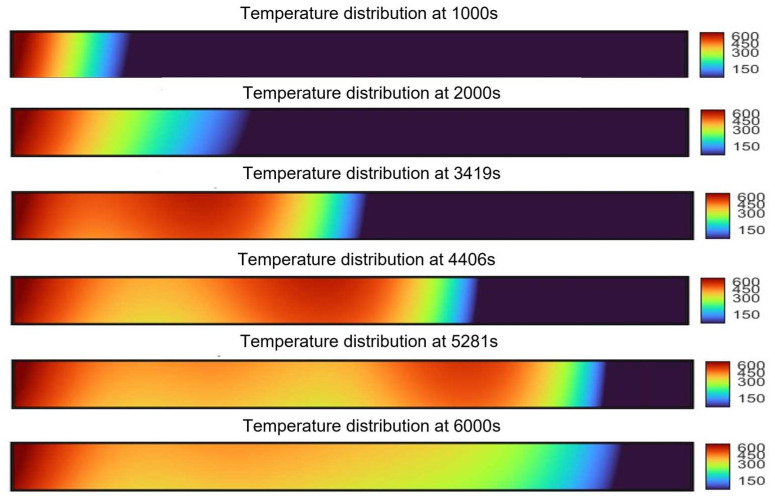
Temperature distribution along the tube at different times.

**Figure 14:**
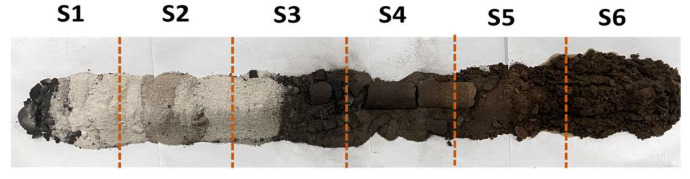
Morphological characteristics of oil sand and sampling points for residual HPAM gel analysis.

Combustion tube experiments were conducted to simulate in situ combustion for enhanced oil recovery in post-polymer-flooded reservoirs. The experimental setup featured a reservoir permeability of 65 $\times {\;10}^{-3}$ μm^2^ and a porosity of 32.2%, with a residual polymer content of 100 μg/g after polymer flooding. The ignition temperature was set at 600 °C. The stable propagation of the combustion front was further evidenced by consistently maintained COx generation levels in effluent gas concentrations, as shown in Figure 12, with an average value of 8.7%. Figure 12 also presents the experiment’s differential pressure and oil recovery factor curves. Figure 13 shows the temperature profiles at different locations along the combustion tube, showing peak temperatures of 502.7 °C (T3), 482.2 °C (T4), 441.6 °C (T5), and 81.7 °C (T6), indicating stable combustion front propagation toward the production well. At 1500 s, as the combustion front neared temperature measurement well T2, a notable increase in the pressure differential commenced. This phenomenon served as a clear indication of the formation of an oil bank, highlighting a critical stage in the combustion process. The maximum differential pressure of 1947 kPa occurred at 2741 s, coinciding with rapid oil production at the production well. The ultimate recovery factor was calculated to be 68.6%. Figure 14 shows the morphological characteristics of oil sand post-ISC. To determine residual HPAM in post-ISC oil sand, triplicate sampling and testing were conducted at six designated points indicated in the figure, ensuring measurement accuracy. The HPAM gel removal efficiency results are presented in Figure 12.

Notably, the highest HPAM gel degradation efficiency of 85.3% was recorded at well T1, in stark contrast to the lowest value of 32.1% observed at well T6. The data obtained reveal a clear inverse relationship between the HPAM degradation efficiency and the distance from the injection well. This correlation aligns with the gradual temperature decrease in the combustion front along the tube. It can be attributed to the enhanced thermal decomposition of HPAM gel at elevated temperatures, which in turn leads to a reduction in residual polymer concentrations. Even though T6 was characterized by low temperature, the continuous generation of high-temperature gases and the movement of crude oil during the ISC process enabled the partial extraction of HPAM through the production well. As a result, despite the incomplete advancement of the combustion front to the production wellbore, the removal efficiency of HPAM at T6 remained above 30%.

Combustion tube experiments showed that HPAM gel had an average degradation efficiency of 64.8% and enabled an oil recovery factor of 68.6%, far exceeding most lab-scale ISC results. These findings indicate that during the ISC process, HPAM gel serves dual functions. On the one hand, it acts as a fuel, promoting the enhancement of crude oil recovery during the process. On the other hand, under the high-temperature conditions of the reservoir and during the secondary displacement process, HPAM undergoes a high efficiency of degradation, effectively eliminating HPAM gel contamination in the subsurface reservoir.

### 2.4. Further Discussion

**Figure 15:**
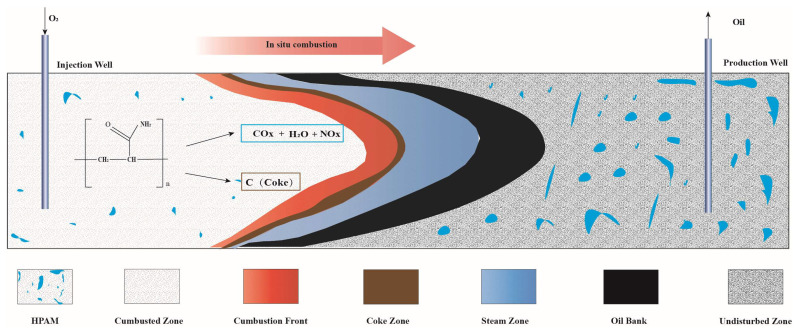
The EOR mechanisms through ISC in polymer gel-treated reservoirs.

Figure 15 provides a schematic representation of the EOR mechanisms through ISC in polymer gel-treated reservoirs.

During the ISC process, distinct thermal zones typically develop within the formation, sequentially including the combusted zone, combustion front, coke zone, steam zone, oil bank, and undisturbed zone [32]. In the combustion zone, residual HPAM gel undergoes substantial thermal degradation. The combustion front is where pyrolysis reactions predominantly occur. At the critical temperature threshold of around 300 °C, the gel first undergoes oxidative carbonization to form coke deposits. This carbon-rich residue serves as essential fuel for sustaining the stable propagation of the combustion front. Subsequently, the increasing temperature promotes further cracking reactions, where HPAM gel decomposes into carbon oxides, water, and nitrogen oxides. These gaseous products combine with hydrocarbon oxidation byproducts to form the steam zone, effectively displacing crude oil toward production wells while remaining largely sequestered in the formation due to flow resistance. The mobilized oil accumulates in the oil bank region, exhibiting reduced viscosity and enhanced mobility. Partial dissolution of combustion gases into the oil phase facilitates production recovery. The undisturbed zone retains both original reservoir fluids and undegraded HPAM gel residues.

This work demonstrates that residual HPAM gel performs dual functions in ISC processes: (1) As a supplemental fuel source through controlled degradation, it enhances the stability of the combustion front and improves sweep efficiency; and (2) in situ decomposition addresses subsurface polymer contamination while eliminating pore-blocking residues. The generated gases contribute to oil displacement while remaining geologically sequestered, presenting an environmentally sustainable EOR approach without additional ecological burdens.

Although the ISC following polymer gel application demonstrates predictable recovery improvement [33], this work analyzes the role of polyacrylamide gel during ISC from a reaction kinetics perspective. The application of ISC in polymer gel-treated reservoirs remains a complex engineering challenge, which requires further investigation into the relationship of the EOR effect with the reservoir characteristics, including porosity, permeability, oil saturation, and so on. Future research should prioritize developing predictive models that integrate chemical kinetics with reservoir engineering principles for comprehensive process optimization.

## 3. Conclusions

The thermochemical degradation behavior of residual HPAM during ISC in post-polymer flooding reservoirs was systematically investigated in this work, and its synergistic effects on EOR and reservoir remediation were studied. Key findings are as follows.

1. Residual HPAM undergoes effective thermochemical degradation during ISC, acting as an in situ fuel to accelerate crude oil oxidation. Quantitative analysis shows that HPAM reduces the activation energy by up to 58.4% in the LTO stage and 75.2% in the HTO stage.
2. ISC achieves dual benefits: it removes residual HPAM while enhancing oil recovery. Experimental results demonstrate an average HPAM removal efficiency of 64.8% across the reservoir, with incremental oil recovery by 68.6%.
3. High-viscosity oil, low-molecular-weight HPAM, and anionic HPAM facilitate HPAM degradation during ISC, with the degradation efficiency ranging from 85.2% to 93.1% under different conditions.

These findings confirm that ISC is a viable follow-up EOR technique for post-polymer flooding reservoirs, offering a sustainable solution to mitigate permeability damage from residual HPAM while boosting oil recovery.

## 4. Materials and Methods

### 4.1. Materials

The materials used in the experiment consisted of HPAM gel and oil sand mixtures, which simulated the porous medium reservoir environment. The oil sand mixtures were prepared via uniform mixing of quartz sand and crude oil. Table 6 and Table 7 show different types of HPAM and crude oil with different viscosities used in the experiments.

**Figure 16:**
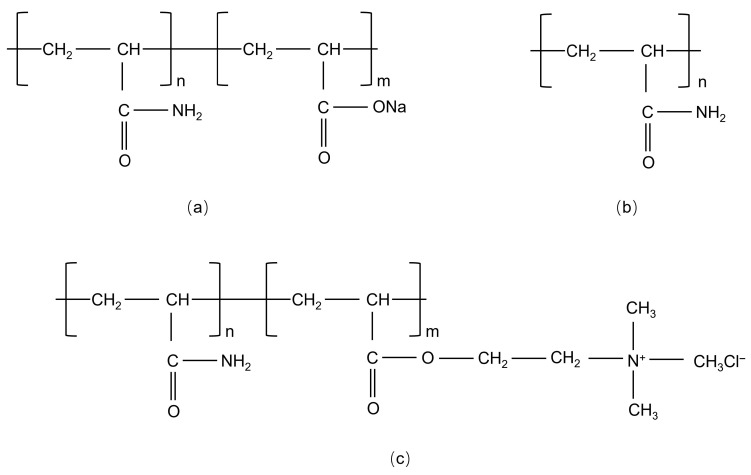
Molecular structure diagrams of anionic polyacrylamide (**a**), nonionic polyacrylamide (**b**), and cationic polyacrylamide (**c**).

All HPAMs used in this work are derivatives of polyacrylamide (repeating unit: –CH_2_–CH(CONH_2_)–) with different charge properties, and their chemical structures are shown in Figure 16. The charge origins are clarified as follows.

The chemical structure of AP contains two main repeating units: neutral amide units (–CH_2_–CH(CONH_2_)–) and anionic carboxylate units (–CH_2_–CH(COO^−^)–). The negative charge originates from the hydrolysis of amide groups (–CONH_2_) in PAM. Cationic units were based on the PAM backbone; AP was modified by introducing cationic monomer units, including Methacryloxyethyltrimethyl ammonium chloride (C_9_H_18_ClNO_2_). The positive charge was derived from the quaternary ammonium groups (–N^+^(CH_3_)_3_) in the modified monomer. The chemical structure of NP was dominated by neutral amide units (–CH_2_–CH(CONH_2_)–) with almost no ionizable groups.

To objectively evaluate the combustion characteristics of HPAM and crude oil during the ISC process without additional catalytic effects, the gels used in the combustion cell experiments and combustion tube in this work are physical HPAM gels with concentrations of 5% and 0.5% (*m*/*v*), respectively. The preparation method was as follows: Prepare 100 mL of deionized water, stir at low speed, and slowly add 5 g/0.5 g of HPAM powder simultaneously. Stir continuously for 12 h to achieve complete dissolution. After stopping stirring, stand them at room temperature for 24–48 h to allow the molecular chains to fully extend and entangle, forming a uniform gel.

### 4.2. Apparatus and Methodology

#### 4.2.1. Reaction Kinetic Experiment

**Figure 17:**
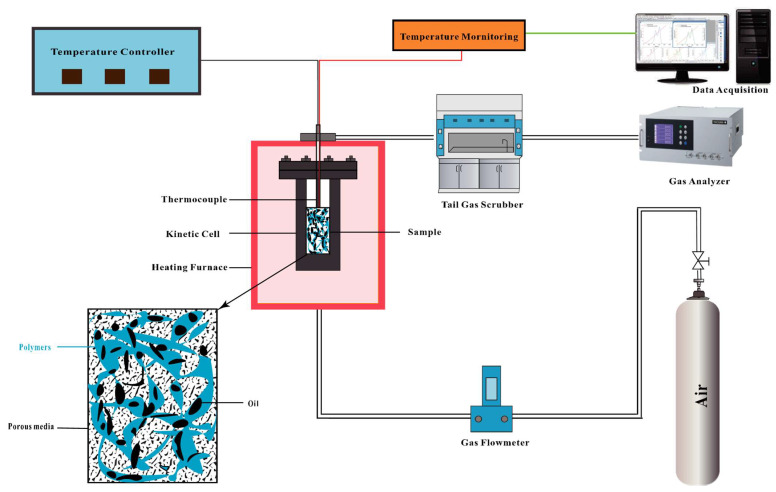
Schematic diagram of the kinetic cell experimental process.

**Figure 18:**
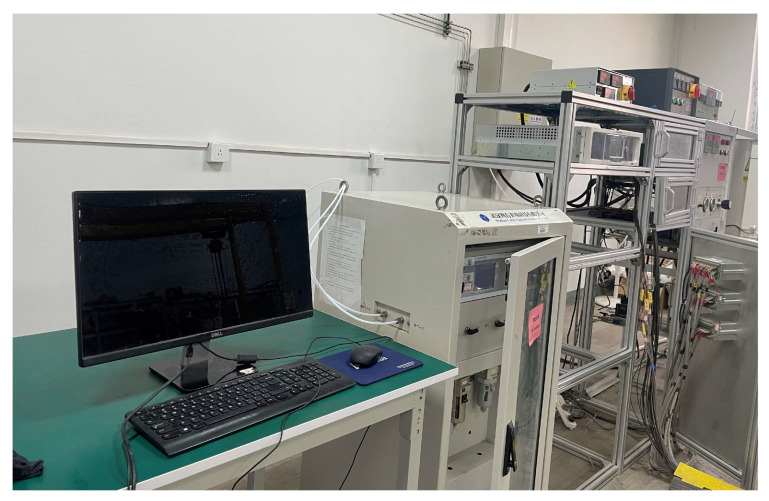
Photograph of the kinetic cell experiment setup.

Kinetic cell experiments were employed to study reaction kinetics and analyze the oxidation behavior of crude oil and HPAM gel within porous media. The schematic of the kinetic cell experimental apparatus is presented in Figure 17. The cylindrical reactor, with a height of 10 cm, features an inner diameter of 2.0 cm and an outer diameter of 3.0 cm. The integrated experimental setup consists of a gas injection system, a temperature-controlled reaction chamber, a temperature detector, a real-time gas concentration monitoring system, a tail gas scrubber, and computerized data acquisition. A detailed photograph of the system is provided in Figure 18.

The experimental design comprised two baseline groups in active energy determination: Group A comprised a porous media system constructed with crude oil to simulate the original reservoir conditions. Group B contained an oil sand system with HPAM gel to simulate post-polymer-flood reservoir conditions. Each experiment of Group A and Group B was repeated with three distinct heating rates (4.1 °C/min, 3.2 °C/min, and 2.6 °C/min) to determine the system’s activation energy.

Parametric studies included Groups C, D, and E, which investigated HPAM gel-containing oil sand mixtures with controlled variables. Specifically, three different molecular weights of HPAM were considered in Group C. Group D evaluated ionic types, including cationic polyacrylamide, anionic polyacrylamide, and nonionic polyacrylamide. Group E maintains a constant anionic HPAM (10–12 MDa) while testing crude oils with different viscosities. The experimental scheme is listed in Table 8 and Table 9.

The experimental procedure is described as follows: The kinetic cell reactor was cleaned and dried. Oil sand samples were prepared: Group A (crude oil: quartz sand = 1:20) and Group B (polymer-contained oil sand = 0.5:1:20), both stirred for 20 min. After placing a filter at the reactor bottom, 5.0 g of quartz sand was loaded. The 10.5 g sample was then added and gently compacted with a glass rod to limit height increase to ≤1.0 cm, followed by 10.0 g quartz sand secured with asbestos. Following gasket sealing and gas line connection, a stepwise pressurization test was set at 2.0 L/min while maintaining a pressure differential below 0.5 MPa. System integrity was confirmed when the stabilized flow rate remained under 0.1 L/min. Air injection commenced, and after >5 min of stable O_2_ concentration, pressures were fine-tuned (differential <0.5 MPa). Heating from 25 °C to 600 °C at a specified rate enabled real-time monitoring of CO_2_/O_2_/CO/CH_4_/H_2_. Heating terminated automatically at 600 °C, with post-cooling disassembly and analysis of temperature/gas concentration data.

#### 4.2.2. Enhanced Oil Recovery Experiment

**Figure 19:**
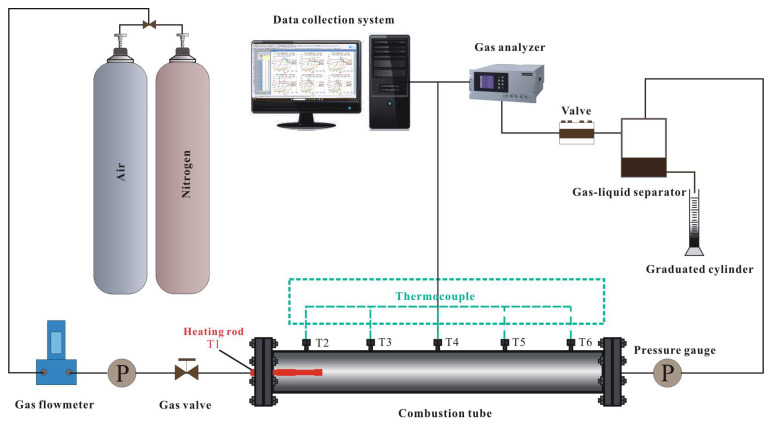
Schematic diagram of the combustion tube experimental process.

The combustion tube experimental setup is a commonly used method for studying in situ combustion EOR techniques. The combustion tube, as shown in Figure 19, was self-designed and comprised an air injection system, an ignition control system, the combustion tube itself, a gas analyzer, and a data monitoring system. The combustion tube was equipped with six temperature-monitoring wells (labeled as T1, T2, T3, T4, T5, and T6), where thermocouples were set inside to monitor temperature variations at different positions.

#### 4.2.3. Activation Energy

Reactant molecules have to overcome a specific energy barrier, known as the activation energy, to engage in effective collisions and then convert into product molecules. In the ISC process, reactants like crude oil molecules and oxygen molecules must acquire adequate energy to leap over this energy barrier, thereby triggering the oxidation reaction. In this work, the activation energy is calculated based on the Friedman formula:

(1) $$\ln\frac{d\alpha }{dt}=-\frac{E_{a}}{RT}+\ln Af(a)$$

where α is the generation concentration of carbon oxides in the effluent gas and is obtained through real-time monitoring by a gas analyzer. *T* refers to the temperature of the reaction cell, which is monitored in real-time by a thermocouple. t is the reaction time and f(a) represents the mechanism function.

Due to the complexity of the reactions of the samples utilized in the experiment, no mature method was established to accurately determine the mechanism function. Therefore, the iso-conversion method was adopted in this work to ascertain the activation energy. Consequently, for a given sample, three sets of combustion cell experiments with different heating rates needed to be carried out. Thus, three curves of $ln\frac{d\alpha }{dt}$ varying with $-\frac{1}{T}$ can be plotted, which are then utilized for the calculation of the activation energy. In total, six sets of experiments were implemented in this part to complete the determination and comparison of the activation energies of the two groups of samples. Specifically, Experiments A1 to A3 (Group A) used oil sand samples, while Experiments B1 to B3 (Group B) used the same oil sand with added residual HPAM. Each experiment was carried out at heating rates of 4.1 °C/min, 3.2 °C/min, and 2.6 °C/min.

#### 4.2.4. Determination of Residual HPAM

A linear relationship (R^2^ > 0.98) was established between concentration (0.1–1.0 g/L) and conductivity of the HPAM gel. Hence, conductivity analysis was implemented for post-combustion residual HPAM gel quantification. The method demonstrated a detection limit of 0.01 g/L with sensitivity <5% RSD. Initially, the standard curves for different types of HPAM were established. HPAM solutions were prepared with concentrations of 0.1 g/L, 0.2 g/L, 0.3 g/L, 0.5 g/L, and 1.0 g/L. Standard HPAM solutions were prepared using deionized water and allowed to equilibrate for 30 min before measurement. Following complete dissolution (magnetic stirring at 500 rpm for 15 min), solution conductivity was measured using a calibrated conductivity meter with temperature compensation. The conductivity data were then calibrated to reference temperature (25 °C) by (2):

(2) $$\rho_{o}=\frac{\rho_{t}}{1+\alpha \left(t_{1}-t_{0}\right)}$$

where $t_{1}$ is the testing temperature, °C $t_{0}$ is the reference temperature 25 °C, and α is the temperature coefficient of the solution, taken as 0.022 here. $\rho_{t}$ and $\rho_{o}$ are the corresponding conductivities at $t_{1}$ and $t_{0}$, respectively.

**Figure 20:**
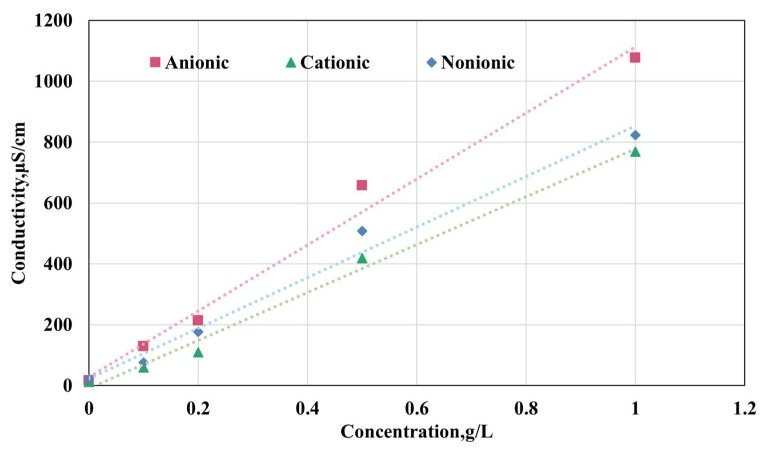
Standard curves of gels with different concentrations.

The experimental measurements were repeated three times to ensure the accuracy and reliability of the data. Subsequently, the average value of the obtained conductivity data was computed and recorded. Based on the validated dataset, calibration curves for cationic polyacrylamide, anionic polyacrylamide, and nonionic polyacrylamide solutions were generated, and they are graphically presented in Figure 20. The corresponding linear regression equations for these curves, which quantitatively describe the relationship between conductivity and concentration, are designated as Equations (3), (4), and (5), respectively.

(3) $$y=787.07x-9.0043\;R^{2}=0.9917$$

(4) $$y=1084.7x+28.368\;R^{2}=0.9871$$

(5) $$y=831.01x+22.436\;R^{2}=0.9848$$

To precisely quantify the residual HPAM gel content in the post-combustion samples, each sample was dissolved in a specific volume of deionized water and continuously agitated for 30 min to ensure complete dissolution of residual HPAM gel. Subsequently, the resultant solution was filtered, and the conductivity of the filtrate was measured using a calibrated conductivity meter. Thereafter, the residual HPAM gel concentration and mass were determined by Equations (3)–(5). The degradation efficiency, denoted as ED, was calculated by the formula presented in Equation (6):

(6) $$ED=\frac{c_{1}\times v_{1}}{m}\times 100\%$$

where $c_{1}$ is the concentration of the residual HPAM after the combustion, $v_{1}$ is the volume of the solution in which the residual sample is dissolved, and m is the original mass of HPAM mixed with the oil sand.

## Figures and Tables

**Table 1:** Calculation results of active energy of oil (Experiment A) in porous media.

Conversion, Fraction	Temperature, °C	Maximum, KJ/mol	Average, KJ/mol
0.005~0.27	268.7~359.5	96.475	54.200
0.27~0.58	359.5~445.1	291.423	210.727
0.58~0.89	445.1~523.5	745.637	239.285
Average	/	/	172.815

**Table 2:** Calculation results of active energy of oil with HPAM gel (Experiment B) in porous media.

Conversion, Fraction	Temperature, °C	Maximum, KJ/mol	Average, KJ/mol
0.005~0.15	242.6~317.3	40.125	24.724
0.15~0.66	337.3~424.6	43.654	34.731
0.66~0.85	424.6~451.7	185.124	63.889
Average	/	/	41.114

**Table 3:** The calculation results of degradation efficiencies for HPAM gel in the experiments C1, C2, and C3.

No.	Experiments	Degradation Efficiency, %
1	C1 (HPAM of 8–10 MDa)	91.5 ± 0.4
2	C2 (HPAM of 10–12 MDa)	89.1 ± 0.2
3	C3 (HPAM of 12–14 MDa)	86.7 ± 0.2

**Table 4:** The calculation results of degradation efficiencies for HPAM gel in the experiments D1, D2, and D3.

No.	Experiments	Degradation Efficiency, %
1	D1 (Cation HPAM)	88.4 ± 0.2
2	D2 (Anionic HPAM)	89.1 ± 0.2
3	D3 (Nonionic HPAM)	87.9 ± 0.1

**Table 5:** Post-oxidation analysis of Experiments E1–E3 quantified HPAM degradation efficiencies.

Experiment NO.	Oil Viscosity, mPa·s	Degradation of HPAM Gel, %
E1	15,310	93.1 ± 0.2
E2	2500	89.9 ± 0.3
E3	128	85.2 ± 0.1

**Table 6:** Characteristics of polyacrylamide polymers with varying properties.

No.	Name	CAS	Manufacturer	Molecular Weight
1	Cationic Polyacrylamide	9003-05-8	Macklin, Shanghai, China	12 MDa
2	Anionic Polyacrylamide	9003-05-8	Macklin, Shanghai, China	12 MDa
3	Nonionic Polyacrylamide	9003-05-8	Macklin, Shanghai, China	12 MDa
4	Anionic Polyacrylamide	9003-05-8	Macklin, Shanghai, China	8–10 MDa
5	Anionic Polyacrylamide	9003-05-8	Macklin, Shanghai, China	10–12 MDa
6	Anionic Polyacrylamide	9003-05-8	Macklin, Shanghai, China	12–14 MDa

**Table 7:** Physicochemical properties of crude oil samples from different oilfields.

Sample No.	Producing Oilfield	Viscosity, mPa·s	Density, g·(cm^3^)^−1^
O1	Nanyang Oilfield	15,100	0.96
O2	Xinjiang Oilfield	1500	0.95
O3	Huabei Oilfield	128	0.90

**Table 8:** Experimental design for Group A and Group B.

NO.	Sample	Ratio (Oil: Sand: [HPAM If Applicable])	Heating Rate, °C·min^−1^
A1	Oil + Sand	1:20	4.1
A2	Oil + Sand	1:20	3.2
A3	Oil + Sand	1:20	2.6
B1	Oil + Sand + HPAM	1:20:0.25	4.1
B2	Oil + Sand + HPAM	1:20:0.25	3.2
B3	Oil + Sand + HPAM	1:20:0.25	2.6

**Table 9:** Experimental design for KC with different HPAM types and crude oils.

NO.	Molecular Weight of Polyacrylamide, MDA	NO.	Type of Polyacrylamide	NO.	Oil Code	Oil Viscosity, mPa·s
C1	8–10	D1	Cationic Polyacrylamide	E1	O1	15,310
C2	10–12	D2	Anionic Polyacrylamide	E2	O2	1500
C3	12–14	D3	Nonionic Polyacrylamide	E3	O3	128

## Data Availability

The corresponding author will provide requested data upon inquiry. For additional details or specific data requests, please directly contact the corresponding author. This work was prepared without the use of AI tools, and the author bears complete responsibility for the publication’s content.

## Abbreviations

The following abbreviations are used in this manuscript:

EOR	Enhanced oil recovery
ISC	In situ combustion
HPAM	Hydrolyzed polyacrylamide
PAM	Polyacrylamide
AM	Acrylamide monomer
LTO	Low-temperature oxidation
HTO	High-temperature oxidation

## References

[1] Juarez-Morejon J.L., Bertin H., Omari A., Hamon G., Cottin C., Morel D., Romero C., Bourdarot G. A new approach to poly mer flooding: Impact of the early polymer injection and wettability on final oil recovery. Proceedings of the SPE Europec featured at 80th EAGE Conference and Exhibition.

[2] Presser D.J., Cafaro V.G., Cafaro D.C. (2021). Optimal Production Strategies for the Development of Mature Oil Fields through Polymer Flooding. Ind. Eng. Chem. Res..

[3] Abedi B., Castano E.P.M., Heidaryan E., Shadloo M.S. (2020). Pore-scale visualization on polymer flooding: Application of singular value decomposition-based image analysis method. J. Porous Media.

[4] Dafaalla M., Azad M.S., Ayirala S., Alotaibi M., Fahmi M., Saleh S., Shehri D.A., Mahmoud M. (2025). Potential of polymer’s viscos ity and viscoelasticity for accessible oil recovery during low salinity polymer flooding in heterogeneous carbonates. Fuel.

[5] Choi B.I., Jeong M.S., Lee K.S. (2014). Temperature-dependent viscosity model of HPAM polymer through high-temperature res ervoirs. Polym. Degrad. Stab..

[6] Beteta A., Sorbie K.S., Johnson G. (2023). Immiscible Viscous Fingering at the Field Scale: Numerical Simulation of the Captain Polymer Flood. SPE J..

[7] Fedorov K., Pospelova T., Kobyashev A.V., Gilmanov A., Kovalchuk T.N., Shevelev A. Determination of Adsorption-Retention Constants and Inaccessible Pore Volume for High-Molecular Polymers. Proceedings of the SPE Russian Petroleum Technology Conference.

[8] Guo H., Wang Z., Dang S., Wen R., Lyu X., Liu H., Yang M. What is Learned from Polymer Flooding Practices in Offshore Reservoirs? In Proceedings of the Offshore Technology Conference, Houston, TX, USA, 1–4 May 2023.

[9] Seright R.S., Wang D., Lerner N., Nguyen A., Sabid J., Tochor R. (2018). Can 25-cp polymer solution efficiently displace 1,600-cpoil during polymer flooding?. SPE J..

[10] Edwards R., Aitkulov A., Redwine C., Cunha K. Viscous oil polymer flood milne point field case history concept to full field implementation. Proceedings of the SPE Improved Oil Recovery Conference.

[11] Thakuria C., Amri M., Saqri K., Jaspers H., Zuhaimi K.H.K. Performance Review of Polymer Flooding in a Major Brown Oil Field of Sultanate of Oman. Proceedings of the SPE Enhanced Oil Recovery Conference.

[12] Yu S., Guo T., Cao G. (2025). Characteristics of sodium p-styrenesulfonate modified polyacrylamide at high temperature under dual scale boundary. Phys. Fluids.

[13] Bao M., Chen Q., Li Y., Jiang G. (2010). Biodegradation of partially hydrolyzed polyacrylamide by bacteria isolated from production water after polymer flooding in an oil field. J. Hazard. Mater..

[14] Hao T., Zhong L., Liu J., Sun H., Zhu T., Zhang H., Wu S. (2022). Mechanistic Study on the Decrease in Injectivity during Salt-Resistant Polymer Flooding. ACS Omega.

[15] Seright R.S., Wang D. (2023). Polymer flooding: Current status and future directions. Pet. Sci..

[16] Gao S. (2014). Influence of The Residual Polymer on The Enhanced Recovery After the Polymer Flooding. Pet. Geol. Oilfield Dev. Daqing.

[17] Miao J., Zhao F., Li S., Yu Y., Chen M. (2005). Presence and distribution of residual polymer in formation. Spec. Oil Gas Reserv..

[18] Stuart M., Lapworth D., Crane E., Hart A. (2012). Review of risk from potential emerging contaminants in UK groundwater. Sci. Total Environ..

[19] Binet S., Bru K., Klinka T., Touzé S., Motelica-Heino M. (2014). Water and acrylamide monomer transfer rates from a settling ba sin to groundwaters. Environ. Sci. Pollut. Res..

[20] Wang X.Y. (2022). Impact of Polymer Flooding on Ecological and Development Environment in Dagang Oil-Field and Its Mechanism.

[21] Sun W., Zhou Q., Wang R., Liu M., Yu W. (2024). Preliminary study on joint degradation mechanism of three poly acrylamide degrading bacteria. J. Yangtze Univ. (Nat. Sci. Ed.).

[22] Andrady A.L. (1994). Assessment of Environmental Biodegradation of Synthetic Polymers. J. Macromol. Sci. Part C Polym. Rev. Part C Polym. Rev..

[23] Xu Z., Ding Y., Tao L., Hu Z., Zhang X., Bai J., Shi W., Li J., Li S. (2024). Low-carbon development strategy to achieve heat conversion in heavy oil reservoirs: In-situ combustion. Geoenergy Sci. Eng..

[24] Zhao R., Yu S., Yang J., Heng M., Zhang C., Wu Y., Zhang J., Yue X.A. (2018). Optimization of well spacing to achieve a stable combustion during the THAI process. Energy.

[25] Deniz-Paker M., Cinar M. (2022). An Experimental Investigation of In Situ Combustion in High Permeability Contrast Systems. J. Energy Resour. Technol..

[26] Steudel A., Friedrich F., Lieske W., Baille W., Konig D., Schuhmann R., Emmerich K. (2019). Simultaneous thermal analysis of cationic, nonionic and anionic polyacrylamide. Heliyon.

[27] Liu J., Zhang B., Yan Z., Xu Z., Zhang Z., Wu X., Wang Q. (2024). Study on thermal decomposition and denitration performance of polyacrylamide. Appl. Chem. Ind..

[28] Zhao R., Wang J., Men Z., He J., Sun Z., Wang T., Li X., Yuan Y., Xu H., Zhang H. (2024). Experimental investigation on cyclic steam stimulation assisted modified THAI to enhance oil recovery in steam-treated heavy oil. Energy.

[29] Silva M.E.S.R., Dutra E.R., Mano V., Machado J.C. (2000). Preparation and thermal study of polymers derived from acrylamide. Polym. Degrad. Stab..

[30] Yang M.H. (1998). The Two-Stages Thermal Degradation of Polyacrylamide. Polym. Test..

[31] Shatat R.S., Niazi S.K., Ariffin A. (2017). Synthesis and Characterization of Different Molecular Weights Polyacrylamide. IOSR J. Appl. Chem..

[32] Guan W., Ma D., Liang J., Li C., Xi C., Zhang X. (2010). Experimental Research on Thermodynamic Characteristics of In-situ Combustion Zones in Heavy Oil Reservoir. Acta Pet. Sin..

[33] Zhao F., Zhu G., Li G., Jiang Y., Liu L. (2022). Feasibility Study on Further Enhanced Oil Recovery by Isc of Remaining Oil after Polymer Flooding. SSRN Electron. J..

